# Space Oddity: microgravity as a neurocognitive catalyst for transformative consciousness experiences

**DOI:** 10.3389/fpsyg.2026.1769177

**Published:** 2026-06-12

**Authors:** Annahita Nezami, Elisa Raffaella Ferre

**Affiliations:** School of Psychological Sciences, Faculty of Science, Birkbeck University of London, London, United Kingdom

**Keywords:** consciousness, gravity, human spaceflight, neuroplasticity, psychedelics

## Abstract

Human consciousness has evolved under the constant pull of terrestrial gravity, yet its role in shaping perception and awareness has received limited theoretical attention. As spaceflight transitions from short missions to long-duration habitation, understanding how consciousness responds to non-terrestrial gravity becomes increasingly urgent. In this perspective, we synthesise behavioural, neurophysiological and neuroimaging evidence to argue that Earth’s gravity functions as a deeply entrenched *1G super-prior* within the brain’s predictive architecture. This super-prior stabilises multisensory integration and constrains large-scale brain network organisation. Exposure to microgravity disrupts vestibular reliability, destabilising this super-prior and triggering cascades of prediction errors that necessitate widespread recalibration across cortical and subcortical systems. We show that these processes extend beyond sensorimotor adaptation, reshaping conscious experience through altered self-location, emotional regulation and perceptual coherence, and potentially underpinning transformative phenomena. Drawing computational parallels with psychedelic states, we propose that microgravity constitutes a non-pharmacological perturbation that transiently relaxes high-level priors, loosens hierarchical constraints and enhances global integration. By situating consciousness in an environment for which evolution offers no precedent, spaceflight provides a unique experiment for probing the contingent foundations of human awareness and the mechanisms through which consciousness can be transformed.

## Consciousness beyond earth’s gravity

Human space exploration is often portrayed as a triumph of engineering, logistics and life-support technology. Yet the most profound consequences of venturing beyond Earth are not mechanical, they unfold within the mind. As lunar bases, commercial spaceflight and Mars missions transition from speculative to operational realities, a pressing question emerges: *how does human consciousness respond when freed from the gravitational anchor that has shaped evolution?* Prolonged exposure to non-terrestrial gravity does more than challenge brain physiology, it reshapes perception, disrupts multisensory integration, induces spatial disorientation, and alters fine-grained sensorimotor control (Goswami et al., 2021; Arshad and Ferré, 2023). In the absence of a stable terrestrial gravitational reference, the brain struggles to maintain reliable estimates of posture, movement, and spatial orientation (White et al., 2020). These perturbations can fragment the continuity of conscious experience, giving rise to subtle depersonalization (detachment from one’s body or thoughts), derealization (the world feels unreal or dreamlike), and even profound blurring of self-environment boundaries (Renaud, 2015). While such effects introduce vulnerabilities, they also open pathways to transformative cognitive and affective states. By perturbing the most stable embodied reference - gravity itself - spaceflight offers a rare window into how fundamental expectations shape consciousness.

Life on Earth has evolved under the constant pull of terrestrial gravity (~9.8 m/s^2^, 1G), a force so pervasive that it is often taken for granted yet profoundly structures human existence. The renowned psychologist Jean Ayres suggested that a child’s relationship to gravity is “even more primal” than its relationship to the mother (Ayres, 1979). A sense of being firmly grounded may underlie all behaviour, carrying deep cultural and existential significance. While one could debate the extent of gravity’s primacy as Ayres proposed, its influence on behaviour surpasses that of any other environmental cue. From foetal development onward, gravity provides a fundamental reference frame for action. Vestibular otoliths, the inner ear receptors that detect linear acceleration and gravity, continuously relay information about head orientation, making gravity the most consistent and reliable sensory signal the brain receives. Bayesian neurocognitive frameworks suggest that predictable sensory input is essential for maintaining a stable, coherent conscious experience (Friston, 2010). In this context, gravity can be seen as an *1G super-prior*: a stable, ever-present reference against which bodily and environmental events are interpreted. During spaceflight, this strong prior conflicts with diminished or absent vestibular signals, generating cascades of prediction errors that the brain must resolve by recalibrating internal models or reweighting sensory inputs (Arshad and Ferré, 2023).

Microgravity disrupts the integration of vestibular, visual, and proprioceptive information (Clément et al., 2020), forcing the sensorimotor system to operate under heightened uncertainty. It also reshapes vestibular-limbic-autonomic coupling (Widjaja et al., 2015; Denise et al., 2007), amplifying emotional reactivity (Rajagopalan et al., 2017), modulating arousal, and altering interoceptive integration (Le Roy et al., 2025). The resulting effects may loosen the boundaries of conscious experience: self-location drifts, bodily coherence becomes less stable, and perceptual integration grows more labile. Astronauts often describe feeling “unmoored,” “expanded,” or “disconnected,” echoing experiences observed in vestibular patients, sensory-deprivation contexts, and certain psychiatric conditions (Sang et al., 2006; Jáuregui-Renaud et al., 2008; Fewtrell and O'Connor, 1988; Elyoseph et al., 2023; Kjellgren et al., 2008; Norlander et al., 2000). Although such alterations can potentially compromise performance and wellbeing, they simultaneously underpin profound transformations in conscious experience. The loosening of perceptual boundaries in microgravity has been proposed as a neurocognitive foundation for the *Overview Effect*: a striking shift in perception, emotion and awareness reported by astronauts when viewing Earth from space. Confronted with the planet’s fragility, unity, and lack of visible borders, astronauts frequently describe an immediate reorganisation of values, heightened ecological concern and a deep sense of interconnectedness with all life (Yaden et al., 2016; Nezami et al., 2021; White, 2021). This transformation reflects a fundamental restructuring of cognitive and affective processing, triggered by a viewpoint for which evolution provided no precedent. As Apollo 14 astronaut Edgar Mitchell famously remarked, “You develop an instant global consciousness… from out there on the Moon, international politics look so petty”.

In this perspective piece, we have reviewed key literature and argued that spaceflight functions both as a perturbation and as a lens, exposing how the brain constructs awareness, maintains continuity of experience and delineates the boundaries between self and world. When Earth-bound constraints are lifted, a rare window opens onto the mechanisms that underpin transformative experience offering insights that are inaccessible under terrestrial gravity conditions.

## Terrestrial gravity as a super-prior: vestibular encoding and Bayesian processing

The vestibular system is the brain’s primary sensor for acceleration and gravity. Rotational accelerations are detected by the semicircular canals, while linear accelerations-including gravity-are encoded by the otolith organs. Together, these receptors transduce physical forces into neural signals, producing a continuous stream of information about self-motion, head position and orientation relative to the environment. Vestibular afferents ascend through hierarchical pathways to reach multiple levels of the nervous system. These include brainstem nuclei that regulate cardiovascular responses and postural reflexes; subcortical structures such as the thalamus, amygdala, and hippocampus, which support affective regulation, spatial memory, and contextual mapping; and cortical regions, including the insula, parietal cortex, and prefrontal areas that underpin. Conscious self-motion perception, interoceptive awareness, and executive control (Dieterich and Brandt, 2015). The vestibular system is unique among sensory modalities in that no primary, unimodal vestibular cortex has been identified in the mammalian brain. Instead, electrophysiological studies reveal multimodal neurons responsive to vestibular, visual, somatosensory, and proprioceptive inputs (Guldin and Grüsser, 1998). Guldin and Grüsser (1998) identified the parieto-insular vestibular cortex (PIVC) as a central hub of the cortical vestibular network. In humans, homologous vestibular activations are distributed across the posterior and anterior insula, superior temporal gyrus, and inferior parietal lobule (Bense et al., 2001; Bottini et al., 1994, 1995; Fasold et al., 2002). Additional vestibular projections extend to primary and secondary somatosensory cortices (Fasold et al., 2002), as well as motor and premotor regions (Bense et al., 2001; Fasold et al., 2002). This distinctive anatomical architecture enables the integration of vestibular signals with visual, proprioceptive, tactile, and visceral information, together with stored knowledge and motor predictions, to construct an optimal representation of the body in space (Ferrè and Haggard, 2020).

At the core of vestibular-multisensory integration lies the computation of an internalised model of gravity, established through lifelong experience with Earth’s gravitational environment (Jörges and López-Moliner, 2017; Lackner and DiZio, 2005; Lacquaniti et al., 2015). Within contemporary Bayesian frameworks, perception emerges from the integration of priors - the brain’s expectations shaped by past experience - and likelihoods derived from current sensory evidence (Friston, 2010). These signals are weighted by their reliability, such that more precise, low-noise inputs exert greater influence on perceptual inference. Because terrestrial gravity represents an exceptionally stable environmental regularity, the internal model of gravity functions as a 1G super-prior: a deeply entrenched expectation that constrains predictions about how bodies and objects behave. Humans are exquisitely adapted to terrestrial gravitational constraints: this super-prior supports a wide range of behavioural functions, enabling accurate anticipation of object motion, efficient navigation of complex environments, and precise prediction of the sensory consequences of action (Brenner et al., 2022; Werkhoven et al., 1992; Zago et al., 2004, 2008). These behaviours illustrate how deeply gravity is embedded in perceptual inference and action planning, serving as a foundational reference against which sensory evidence is interpreted. In microgravity, this inferential architecture is profoundly disrupted. Vestibular signals lose precision, visuomotor contingencies become unreliable, and prediction errors accumulate, destabilising the brain’s hierarchical predictive model. Under these conditions, the brain increasingly relies on bottom-up sensory inputs while system-wide recalibration unfolds across subcortical circuits and multimodal cortical networks. Sensory integration becomes less optimal, and internally generated signals may be misattributed as external. Together, these processes underlie phenomena such as space adaptation syndrome and the perceptual disorientation commonly reported by astronauts (Khalid et al., 2023).

From a computational standpoint, microgravity perturbs the delicate balance between priors and sensory evidence. The 1G super-prior loses reliability, necessitating dynamic adjustments in the weighting of vestibular, visual, and proprioceptive inputs. Simultaneously, microgravity diminishes perceptual suppression through weakened GABAergic inhibition and altered thalamo-cortical gating, thereby enhancing sensory gain and multisensory integration (Fox et al., 1997). Vestibular-autonomic coupling is disrupted, heart rate variability becomes fragmented, and the sympathetic-parasympathetic balance shifts (Blaber et al., 2010; Benítez-Salgado et al., 2024). These physiological perturbations manifest as emotional lability, altered arousal states and sleep disturbances, reflecting ongoing recalibration rather than pathology. Collectively, these changes destabilize the predictive hierarchy, loosening perceptual and bodily boundaries and triggering a profound recalibration of spatial and self-related representations. As a result, conscious experience can be fundamentally transformed. Astronauts frequently report depersonalization/derealisation-like sensations, heightened sensory awareness, and intensified introspective insight - phenomena such as the Overview Effect - reflecting the brain’s continuous effort to reconcile conflicting priors with incoming sensory information. These observations illustrate that extreme disruption of a deeply entrenched super-prior can reshape cognition in ways unattainable under terrestrial gravity conditions.

## Disrupting the 1G super-prior: microgravity and the opening of consciousness states

Spaceflight induces profound structural and functional changes in the human brain. Structural magnetic resonance imaging (MRI) studies comparing astronauts before and after long-duration missions have consistently reported a redistribution of subarachnoid cerebrospinal fluid (CSF), ventricular enlargement and macroscopic gray matter modifications indicative of shape changes and tissue remodelling (Van Ombergen et al., 2017a, 2017b; Wuyts et al., 2025). These alterations are largely attributed to the upward shift of bodily fluids upon exposure to microgravity (Wang et al., 2026). Increases in neural tissue have been observed in several sensorimotor regions, including the cerebellum and basal ganglia, pointing toward neuroplastic adaptations that support motor strategy recalibration in response to altered gravitational constraints (Van Ombergen et al., 2017a, 2017b; Wuyts et al., 2025). Functionally, resting-state fMRI studies have revealed widespread connectivity changes in response to microgravity (Van Ombergen et al., 2017c; Pechenkova et al., 2019; Jillings et al., 2023; Doroshin et al., 2022). A single-cosmonaut study demonstrated that vestibular regions showed reduced participation in whole-brain functional connectivity following 169 days in microgravity (Demertzi et al., 2016). Subsequent group-level studies have extended these findings, revealing that vestibular, motor, and multisensory integration regions, including the supramarginal gyrus, posterior and anterior insula, angular gyrus and temporo-parietal junction, undergo significant alterations in whole-brain connectivity (Jillings et al., 2023). These changes likely reflect adaptive reweighting of sensory inputs and recalibration of sensorimotor predictions. Notably, postflight increases in visual and somatosensory activations in response to vestibular stimulation have been observed, with recovery occurring over the months following return to Earth, highlighting the dynamic and plastic nature of these adaptations. The posterior cingulate cortex (PCC), a central hub of the default mode network (DMN), shows reduced global connectivity after spaceflight, a reduction that can persist for months (Jillings et al., 2023). Given the PCC’s role in arousal, awareness, integration of internal and external stimuli and detection of environmental changes, these findings suggest that it contributes to adaptive reorganization in response to unfamiliar microgravity input. Electrophysiological measures further support these findings. Resting-state EEG analyses focusing on DMN alpha-band power which reflects oscillatory activity in the 8–12 Hz range associated with cortical inhibition, attentional regulation and top-down control and functional connectivity reveal significant decrease during and after spaceflight, with reductions persisting for up to 20 days post-landing (Pusil et al., 2023). These changes indicate electrocerebral alterations that may serve as a neurophysiological marker of functional brain adaptation during space missions.

Mechanistically, the structural and functional changes observed in astronauts can be understood through the lens of the 1G super-prior. Gravity is among the most stable and deeply entrenched references in human experience, shaping perception, motor control, spatial orientation, and higher cognition. In the microgravity of spaceflight, this fundamental super-prior no longer aligns with the diminished or absent vestibular otolith signals, triggering cascades of prediction errors that the brain resolves by recalibrating internal models or reweighting sensory inputs. The resulting relaxation of top-down constraints opens a rare window for neuroplasticity, enabling widespread reorganization across cortical and subcortical networks and revealing latent capacities for perception, cognition, and consciousness. Remarkably, these adaptations echo neural phenomena observed under psychedelic compounds. Psilocybin and LSD increase global functional connectivity, weaken canonical hierarchical networks, and reduce the precision of high-level priors, producing alterations in self-boundaries and conscious awareness (Siegel et al., 2024; Bedford et al., 2023; McCulloch et al., 2022). For example, psilocybin decreases connectivity within high-level associative networks while enhancing connectivity in sensory areas, generating a global re-integration pattern characteristic of the psychedelic state (Siegel et al., 2024). Neuroimaging further shows that psychedelics flatten the functional hierarchy, reducing the canonical distinction between unimodal (sensory/motor) and multimodal (associative/self-referential) cortical regions (Siegel et al., 2024).

Analogous patterns emerge in astronauts exposed to microgravity. Resting-state fMRI reveals reduced DMN dominance, altered participation of unimodal and multimodal sensory hubs, and persistent changes in whole-brain connectivity (Demertzi et al., 2016; Jillings et al., 2023). Reductions in EEG alpha-band power mirror findings from psychedelic states, where decreased alpha activity correlates with elevated neural entropy and altered self-perception (Carhart-Harris et al., 2016; Klimesch, 2012). Although the initiating perturbations differ - pharmacological serotonergic modulation versus environmental gravity disruption - the computational consequences converge: high-level priors are transiently relaxed, canonical network hierarchies loosened, and global integration enhanced. Network control theory characterizes this as a reduction in the brain’s “control energy landscape”, lowering the energetic cost of transitions between states and enabling exploration of a broader repertoire of network configurations (Singleton et al., 2022). These dynamics, coupled with enhanced bottom-up integration, underpin hallmark phenomena observed under psychedelics, including ego-dissolution, altered perception and expanded conscious experience.

**Figure 1:**
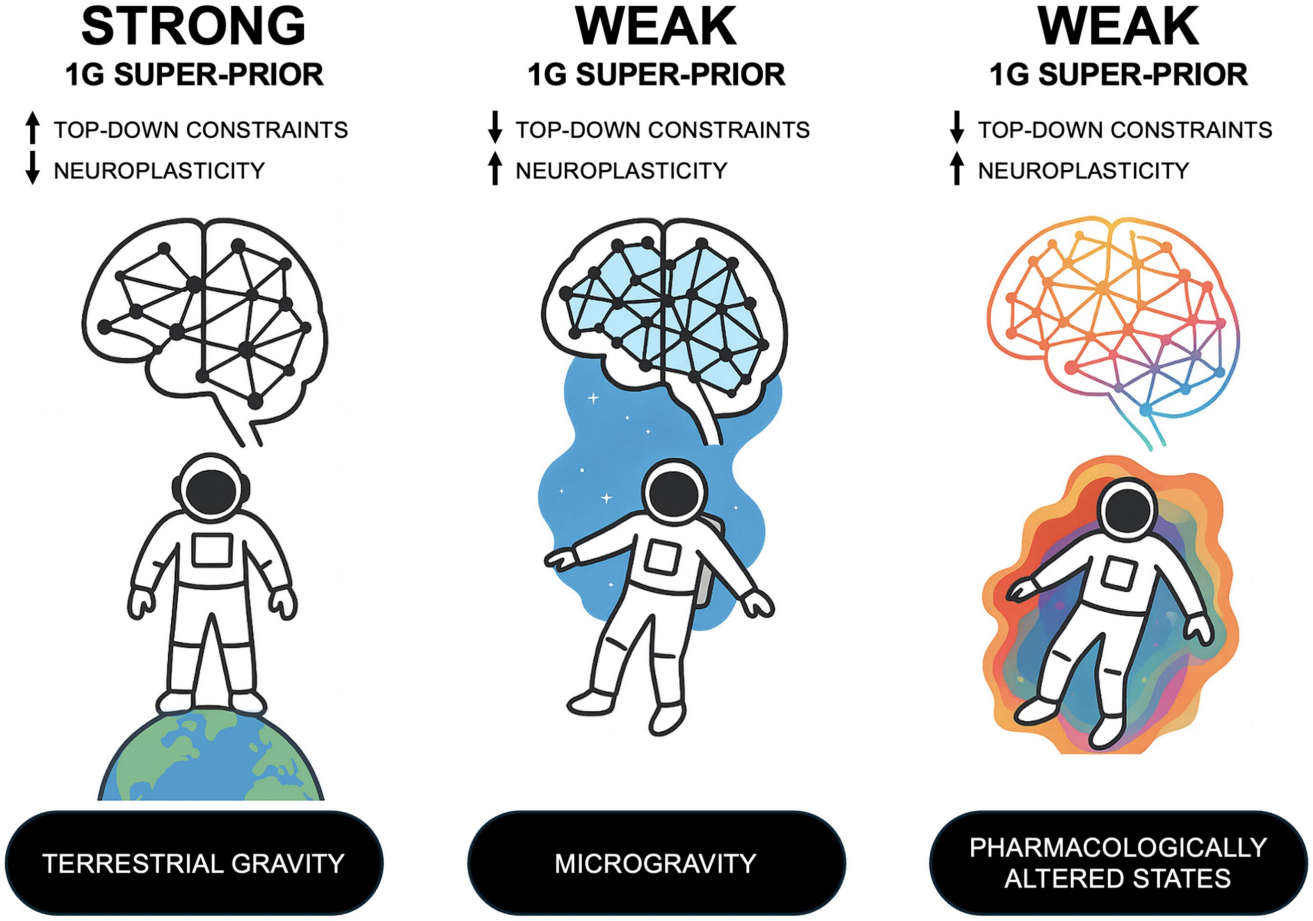
Comparison of brain network organization and 1G super-prior strength across terrestrial gravity, microgravity, and pharmacologically altered states. Weakening the 1G super-prior whether via environmental or neurochemical perturbation reduces top-down constraints and increases neuroplasticity, enabling expanded perceptual and cognitive states.

Microgravity therefore represents a non-pharmacological analogue of the neural “unlocking” observed under psychedelics (Figure 1). By attenuating top-down precision and enhancing sensory gain, spaceflight permits access to cognitive and perceptual states that remain largely inaccessible under Earth-bound conditions. Crucially, the parallels between microgravity and psychedelic states should not be taken to imply mechanistic equivalence. Psychedelic compounds produce altered states primarily via pharmacological modulation of serotonergic receptors, particularly 5-HT2A, resulting in widespread changes in cortical excitability and synaptic gain. In contrast, microgravity constitutes an environmental perturbation that selectively diminishes vestibular reliability and reshapes multisensory prediction error signalling. The convergence between these states lies not at the receptor or neurochemical level, but at the computational level: in both cases, the precision of deeply entrenched high-level priors is transiently reduced, canonical network hierarchies are relaxed, and large-scale integration is enhanced. This distinction is critical, framing microgravity-induced alterations not as drug-like states, but as environmentally driven reorganizations of predictive inference that reveal the latent plasticity and flexibility of human perception, cognition, and consciousness (Figure 1).

## Conclusive remarks

Taken together, the evidence reviewed here supports a simple but far-reaching claim: gravity is not merely a physical constraint on the body, but a foundational scaffold for human consciousness. Terrestrial gravity operates as a deeply entrenched super-prior that stabilises perception, anchors the sense of self, and constrains the brain’s predictive architecture. When this reference is disrupted in microgravity, the consequences extend well beyond sensorimotor adaptation. Prediction hierarchies loosen, multisensory integration is reweighted, and large-scale brain networks reorganise, creating conditions in which conscious experience itself becomes more fluid, labile and expansive. Microgravity thus exposes a latent flexibility in the human brain. By selectively weakening one of the most reliable expectations shaped by evolution, spaceflight reveals mechanisms that remain largely inaccessible under Earth-bound conditions. The resulting phenomena - ranging from spatial disorientation and depersonalisation-like experiences to heightened introspection and the Overview Effect - should not be viewed as anomalies. Rather, they reflect the brain’s active effort to recalibrate its most fundamental assumptions about the body, space and the self in the absence of a stable gravitational reference. In this sense, microgravity acts as a perturbation that renders visible the normally hidden operations of predictive inference that sustain continuity of experience on Earth. Parallels with psychedelic states further illuminate this process. Although microgravity and psychedelics differ profoundly in their proximal causes, both converge at a computational level by transiently reducing the precision of high-level priors, loosening canonical network hierarchies and enhancing global integration. These shared dynamics underscore a general principle: transformative states of consciousness emerge when deeply entrenched expectations are relaxed, allowing alternative modes of perception, affect and self-representation to unfold. Spaceflight thus offers a rare, non-pharmacological model for studying how consciousness reorganises when its most stable constraints are lifted.

By situating consciousness in an environment for which evolution offers no precedent, spaceflight positions microgravity as a powerful natural neurocognitive catalyst for transformative experience. It reveals how expectation, embodiment and environmental structure jointly co-produce the architecture of the human mind. As human presence in space expands toward longer missions and planetary habitation, understanding these processes becomes critical, not only for safeguarding astronaut wellbeing and performance, but also for advancing a deeper scientific account of consciousness itself. In stepping beyond Earth’s gravity, we gain a unique vantage point from which to examine the contingent foundations of human awareness and the conditions under which it can be fundamentally transformed.
